# Overweight and Obesity among Preschool Children from Fars Province of Iran: Prevalence and Associated Factors 

**Published:** 2016-02-17

**Authors:** Zahra Hassanzadeh-Rostami, Elham Kavosi, Aliasghar Nasihatkon

**Affiliations:** ^a^ Department of Community Nutrition, Nutrition and Food Sciences Research Center, School of Nutrition and Food Sciences. Shiraz University of Medical Sciences, Shiraz, Iran; ^b^ Department of Nutrition, Shiraz University of Medical Sciences, Shiraz, Iran; ^c^ Fars Petroleum Industry Health Organization, Fars, Iran

**Keywords:** Overweight, Obesity, Children, Preschool, Prevalence

## Abstract

**Background:** The global prevalence of overweight and obesity had risen in recent decades, and obesity is taken into consideration as a public health concern and a major risk factor of common chronic disease. The objective of the present study was to estimate the prevalence of overweight and obesity and to identify its underlying factors among children 2-6 yr of age in Fars Province, southern Iran.

**Methods:** A total of 8911 children, aged 2-6 yr age, were selected thorough multi-stage sampling in 30 cities of Fars Province in 2012-13. Overweight and obesity status was assessed through comparison by standard BMI for age and for sex percentiles (NCHS/CDC). In addition, socio-demographic measures obtained from structured questionnaire were compared between normal and overweight plus obese (ow/ob) groups. We used backward stepwise Logistic Regression, Chi-square and Independent sample t-test to relate the underlying factors to the nutritional indices.

**Results:** The prevalence of overweight and obesity was 5.7% (95% CI: 5.2%, 6.1%) and 5.2% (95% CI: 4.7%, 5.6%), respectively. The ow/ob was significantly correlated with male sex, urbanization, type of father’s occupation, universal education of mothers, the higher birth weight, low access to household facilities, and not using health-care services (P<0.05). Besides, the logistic regression analysis showed urbanization (OR=1.46, CI: 1.26, 1.70), second sibling (OR=1.183, CI: 1.00, 1.39), and less access to a variety of food groups (OR=1.32, CI: 1.05, 1.65) as ow/ob risk factors.

**Conclusions:** The rate of overweight and obesity in the study population is at an alarming level. Therefore, a preventive program is needed to control ow/ob since early childhood considering the underlying factors for each region and individual groups.

## Introduction

Worldwide, the prevalence of overweight and obesity (ow/ob) is increasing and has become a serious public health concern^1,2^. Although childhood obesity is more prevalent in developed countries as an epidemic situation, in recent years, the rate of obesity in children and adolescents in developing countries is also increasing; it is the consequence of economic and cultural development and nutrition transition^1,3^. On the other hand, obesity is also in a high level in countries that experience protein- energy malnutrition and are confronted with a “double burden” of nutrition-related health problems^1,3^.

In 2010, WHO has reported globally 6.5% of children are overweight and this rate has been increased approximately 2% compared to 1990 ^4^. Besides, this rate among Asian children was 4.6% ^4^. Furthermore, the prevalence of overweight in children under 5 years of age in Eastern Mediterranean Regions has shown a range of 2.3% to 17.5%^5^. The worldwide rate of overweight in preschool children will increase to 11% by 2025, affecting 70 million children^1^.

Like other areas, Iran has shown a rising trend in childhood overweight in recent years and a wide range of overweight in preschool children from 3% to 24.8% and obesity from 3.8 to 8% has been reported (1995-2011)^6-12^.

Environmental factors have shown substantial effects on the obesity pattern. In this issue, modernization and economic development along with urbanization are important factors, which can affect the standard of living and services available to people ,^3^. These factors change the lifestyle, affect the food habits and physical activity of children, and expose them at the risk of overweight and obesity^1,3,5^.

Obesity has psychological consequences including social isolation, low self-esteem and depression that may threaten child and community health ^1^. In addition, childhood obesity increases the risk of adult obesity and its related common diseases such as hypertension, myocardial infarction, glucose intolerances, asthma, and other respiratory problems, sleep disorders, liver disease, infertility, cancers, orthopedic disorders, etc. ^1^. Moreover, WHO had considered non-communicable diseases as a main cause of 3 quarters of deaths in 2020, in developing countries ^1,3^.

Due to the seriously increasing trend of ow/ob in children and adolescents of most communities, it is important to control this nutritional disorder at early years of age with identification of its underlying factors. In this regard, we carried out this study to determine the prevalence and risk factors of overweight and obesity in children 2-6 years of age in Fars Province, Iran.

## Methods

We used the eligible data of a cross-sectional survey performed in Fars Province of Iran during December 2012 to January 2013 ^13^. The study subjects were 8911 preschool children aged 2-6 yr selected through door-to-door visits. The interview team informed the parents or child’s legal caregiver about the study goals and then obtained verbal consent. Inclusion criteria were healthy children aged 2–6 yr, and the exclusion criteria were those with no chronic disease, metabolic disorders, and any drug usage affecting the child’s growth.

Anthropometric measurements were done by trained health care staff. Body weight was measured by a digital weighing scale (Seca, Germany) with the margin error of 0.1 kg. The height or length were measured by a measuring board for babies and toddlers (Seca, Germany) with the margin error of 0.1 cm. Subjects were wearing light clothes and were bare-footed through the measurements. We used NCHS/CDC cut-off points to determine overweight and obesity. Body mass index (BMI) was computed as the ratio of the weight in kg to the height squared in meter. The BMI between 85 and 95 percentiles for age and sex was defined as overweight and the BMI greater than 95 percentiles for age and sex was defined as obese.

To gather demographic and socio-economic data, we used a structured questionnaire including child age and gender, child birth weight (recorded from the child health report card), birth order, number of younger siblings, family size, child’s care-giver, household head, parental education and occupation, family income (categorized in 5 levels; lower than 134 US dollars, 134–200 US dollars, 201–334 US dollars, 335–667 US dollars, and higher than 667 US dollars), house meter, type of settlement, religion, ethnicity, health services usage, access to safe water supply, and household facilities and furniture including access to freezer, washing machine, dish washing machine, microwave, computer, cell phone, and personal car. Then we compared the underlying factors between normal and ow/ob groups.

Statistical analyses were done using the SPSS software package, version 16.0 (SPSS Inc., Chicago, IL, USA). ow/ob were determined by the Epi-Info software program, version 2008 for Windows. Backward stepwise Logistic Regression analyses have been used to relate the underlying factors to the nutritional indices. Independent sample *t*-test and Chi-square test were also used to determine the differences in variables between the normal and ow/ob groups. Values are expressed as mean ± SD (for numerical data) and N (%), (for categorical variables). A *P*<0.05 was indicated statistically significance in all statistical tests.

## Results

This study was performed on 8911 children between 2-6 yr of age, of whom, data of 90 subjects were missed because of incomplete questionnaires. The number of males and females was 4618 and 4203, respectively. 48.0 and 52.0% of the subjects resided in urban and rural regions.

The prevalence of overweight (BMI between 85-95 percentiles for age and sex), obesity (BMI ≥95 percentile for age and sex), and underweight (BMI ≤5 percentile for age and sex) was 5.7, 5.2, and 25.3%, respectively. Therefore, 63.9% of the children were categorized as normal group.

After removing underweight subjects, the baseline characteristics were measured (Table 1) and the differences between normal and ow/ob groups were analyzed. The socio-economic and family related determinants of overweight and obesity are reported in Table 2. As shown, the rate of ow/ob in boys was significantly higher than girls was (*P*=0.048). In addition, children residing in urban area were significantly heavier than those in rural areas were (*P*=0.001). Moreover, children who used health-care services were less overweight or obese than the others, i.e., 14% vs. to 19% (*P*=0.026). Our results did not show any remarkable association between ow/ob and each years of age. We found the universal education of mothers as the risk factor of ow/ob (*P*=0.01). In addition, children whose fathers were jobless, farmer, and worker were less ow/ob compared to others (*P*=0.013). Moreover, being heavier at birth time and lower accessibility to household facilities were the determinants of childhood ow/ob (*P*<0.05).

**Table 1:** Demographic and socioeconomic characteristics of the study subjects

**Variables**	**Number**	**Percent**
Child`s sex
Male	3501	52.0
Female	3237	48.0
Type of settlement
Urban	3237	48.0
Rural	3501	52.0
Mother's education
Lower than diploma	4122	61.2
Diploma	1840	27.3
Universal education	768	11.4
Father's education
Lower than diploma	4217	62.6
Diploma	1627	24.1
Universal education	839	12.5
Mother's occupation
Housewife	6213	92.2
Working mothers	522	7.8
Father's occupation
Unemployed	236	3.5
Farmer	968	14.4
Worker	1535	22.8
Employee	945	14.0
Retired	65	1.0
Free jobs (Business, etc.)	2949	43.8
Birth order
1^st^	3765	55.9
2^nd^	1905	28.3
3^rd^	681	10.1
4^th^	233	3.5
5^th+^	144	2.2
Family size
1-4	4826	71.6
>4	1906	28.3
Who care the child
Mother	6655	98.8
Others	81	1.2
Birth weight (gr)
<2500	624	9.4
2500-4000	5813	87.9
>4000	175	2.6
Family income
1^st^ level	2605	38.7
2^nd^ level	2463	36.6
3^rd^ level	1268	18.8
4^th^ level	341	5.1
5^th^ level	52	0.8
Ethnicity
Fars	5551	82.4
Ghashghaei	417	6.2
Khamse	21	0.3
Mamasani	440	6.5
Arab	87	1.3
Other	220	3.3
Healthcare services
Yes	6479	96.2
No	253	3.8
Access to household facilities and furniture
0 item	86	1.3
1 item	348	5.2
2 item	964	14.3
3 item	1469	21.9
4 item	1671	24.9
5 item	1429	21.3
6 item	668	9.9
7 item	85	1.3
Food Availability
Yes	5896	87.5
No	823	12.2

Furthermore, we found three more underlying factors of ow/ob from backward stepwise Logistic Regression analysis. Urbanization was determined as ow/ob risk factor (OR=1.46, CI: 1.26, 1.70). Birth rank was another factor affecting nutritional status. The rate of ow/ob in the second siblings was higher than the first children was (OR=1.18, CI: 1.00, 1.39). The OR of ow/ob in children with no access to food groups was 1.32 times the others (OR=1.32, CI: 1.05, 1.65).

## Discussion

The present study revealed the prevalence of ow/ob as 5.7 and 5.2 percentage, respectively in 2-6 yr old children in Fars Province of Iran. To our knowledge, there was no published paper to show the prevalence of obesity in this age group in the province. However, 10.8 and 5.1% of Iranian preschool children were respectively ow/ob in 2000s ^14^. Moreover, Jafari-Adli et al. reported the rate of overweight in the range of 5%-13.5% and obesity in 3.2-11.5% during 2005-2014 ^15^. Although our results are categorized in this range, however, place in the lower limit. Therefore, preschool children in Fars province are at lower rate of overweight and obesity relative to most other part of Iran. In addition, compared to earlier report ^14^, the rate of overweight in Fars Province was in a better situation; however, obesity was still at a remarkable level. Our study-analyzed subjects from urban and rural areas in nearly similar rates, whereas population in some previous studies was only from urban areas, so it can affects the estimated prevalence of overweight and obesity. Furthermore, in this study, the most children were from families with lower levels of income and their parents had lower educational status. Therefore, the lower rate of overweight is probably due to socio-economic inequality.

Recent studies have shown an increasing trend of obesity in all age groups. In Tehran, Iran, also elevated rates of ow/ob have been represented from 3.5% in 1990 to 6% in 2001 and 7.8% in 2013 ,^11^. Moreover, developing countries are faced with a rising rate of obesity from 4.2% in 1990 to 6.7% in 2010 and it is expected to reach 9.1% in 2020 ^2^. The improvement of socioeconomic status during last years in EMR countries has altered diet styles which lead to higher consumption of fats specially saturated fats, cholestrol, refined carbohydrates and less poly-unsaturated fatty acids and fibers. Diet altering together with physical inactivity has increased the risk of obesity. Hence, because of the greater impact of fat and sugar in energy production in higher income conditions, the nutrition transition phenomenon is more noticed in high- and middle- income countries ^5^.

In the second part of the study, we assessed the differences of underlying variables between normal and ow/ob groups. Male sex has been determined as a risk factor of ow/ob, as the boys were significantly heavier than girls were. Several studies have reported different distributions of ow/ob according to gender ^16-18^. In line with our results, mirmiran et al. have expressed higher prevalence of both ow/ob in boys compared to girls in Iran and some other Middle East Countries ^3^. Besides, boys in developing countries and girls in developed countries were more obese ^3^. Furthermore, some studies showed higher rates of overweight in girls and obesity in boys ,^10^. The differences observed in the studies are due to different cultural and environmental factors and food habits. In addition, the people’s knowledge and attitude towards the food patterns and physical activity in addition to their beliefs about child’s nutritional status are mostly the main causes.

Urbanization was another significant factor related to ow/ob. Obesity was more prevalent in urban areas of EMR countries; also, Risia et al. reported more obesity in children residing in urban areas ,^20^. Urbanization is accompanied with reduced physical activity, increased food availability, high consumption of fast foods and junk foods. In addition, with the increase in family income in urban areas, they have spent more leisure time in restaurant and hence take foods with more energy, fat, cholesterol and less fiber. Inconsistently Lutfiyya et al. reported more overweight children in rural areas; however, they also stated that other obesogenic factors such as development indices maybe the causes of childhood overweight in rural residency ^21^.

Parental education and occupation were considered as the underlying factors of ow/ob. Children whose fathers were jobless, farmer and worker were significantly less overweight and/or obese. Well-paying jobs, which in this study are categorized as free jobs, are associated with high-family income and could affect childhood obesity. Nonetheless, we could not find any significant association between family income and ow/ob, although the rate of childhood ow/ob was higher in high-income families (Table 2). Similarly, in recent studies, parental occupation was also determined as the positive risk factor of childhood obesity, due to its high-income acquisition ^22^. However, Thibault et al. presented more obesity prevalence in adolescent whose father’s occupation was categorized in low level of socioeconomic status. These differences may be affected by other contributing factors of obesity ^23^.

**Table 2:** Distribution of overweight/obese in preschool children in relation to socio-economic factors

**Variables**	**Normal**		**Overweight/Obese**		***P*** ** value**
**Categorical**	**Number**	**Percent**	**Number**	**Percent**	
Sex					0.048
Boy	2976	85.0	525	15.0	
Girl	2806	86.7	431	13.3	
Type of settlement				0.001
Urban	2707	83.6	530	16.4	
Rural	3075	87.8	426	12.2	
Age (yr)					0.168
2-2.9	1672	84.5	307	15.5	
3-3.9	1405	86.6	217	13.4	
4-4.9	1380	85.7	231	14.3	
5-6.9	1325	86.8	201	13.2	
Mother's education				0.014
<Diploma	3562	86.4	560	13.6	
Diploma	1581	85.9	259	14.1	
Academic	633	82.4	135	17.6	
Father's occupation				0.013
Jobless	208	88.1	28	11.9	
Farmer	859	88.7	109	11.3	
Worker	1332	86.8	203	13.2	
Employee	796	84.2	149	15.8	
Retired	2497	84.7	452	15.3	
Free jobs	54	83.1	11	16.9	
Family size					0.067
1-4	4117	85.3	709	14.7	
>4	1659	87.0	247	13.0	
Food Availability				0.521
Yes	5064	85.9	832	14.1	
No	700	85.1	123	14.9	
Healthcare services				0.026
Yes	5572	86.0	907	14.0	
No	205	81.0	48	19.0	
Ethnicity					0.068
Fars	4749	85.6	802	14.4	
Ghashghaei	375	89.9	42	10.1	
Khamse	17	81.0	4	19.0	
Mamasani	370	84.1	70	15.9	
Arab	80	92.0	7	8.0	
Other	189	85.9	31	14.1	
Birth weight (gr)					0.017
<2500	545	87.3	79	12.7	
2500-4000	4991	85.9	822	14.1	
>4000	138	78.9	37	21.1	
**Continuous**	**Mean**	**SD**	**Mean**	**SD**	**P value**
Facilities	3.75	1.45	3.62	1.47	0.012
Income	1.92	0.91	1.98	0.93	0.069
House meter	131.37	130.62	140.44	235.57	0.084

Higher education level of mothers was a risk factor of ow/ob. Mothers with universal education significantly had more ow/ob children compared to diploma and lower education levels. Likewise, recent studies ,^24^ have shown similar data. It may be due to more chances for employment in women with higher education level, and so more income. Education level is directly related to socio-economic status. High education and income, at first, results in unhealthy diet and leads to obesity. However, over the years, education can improve knowledge and become a protective factor, as the trend of obesity tends to decrease. Moreover, mothers working out of home have less time to take enough care on nutritional status and physical activity of their children.‏

Higher birth weight as an individual factor was a significant positive predictor of ow/ob and the same results have been shown in the most other studies ,,^25^. However, to our knowledge, inconsistent relation was not seen in literatures.

We observed children who were not monitored in national health-care services centers were significantly more overweight and obese. Therefore, it can confirm the necessity of health enhancing and weight control programs of these centers. Interestingly, the rural subjects were the most clients of health-care centers. As we reported previously, the occurrence of ow/ob in rural areas was significantly lower than urban areas. Furthermore, families with high-income level do not tend to take public health services and skip the routine monitoring programs, so they are more exposed to nutritional disorders. Similarly, Lutfiyya et al. reported higher prevalence of overweight in children with no preventive health care in the past 12 months ^21^.

Families’ accessibility to household facilities was determined as a significant factor affecting childhood nutritional status. The rate of ow/ob was lower in children of families with more access to personal car, computer, microwave, cell phone, dish washing machine, and washing machine. However, previous studies reported a positive association between obesity and household goods, and presented household facilities as an indicator of socio-economic development ,^20^. The previous studies were assessed as basic equipment such as refrigerator, TV, oven, etc., whereas we analyzed items representing high levels of well fare and economic growth. Thus, a little socio-economic development can increase obesity and much more development may correct this situation. However, in the present study, most of the families were not categorized in very high or very low economic levels and probably they paid more for basic living equipment and they could provide daily foods especially low cost and energy dense snakes.

Of all variables in backward stepwise logistic regression model, urbanization, low accessibility to main food groups, and second sibling were the most significant predictors of ow/ob.

Families with easy access to main food groups had healthier dietary pattern and consequently less nutritional disorders including overweight and obesity. Restricted access to food items most probably leads to lower intake of fruits and vegetables and high intake of fat and refined carbohydrates that predominantly cause obesity. In this regard, consumption of empty calories (solid fat and added sugars) accelerate weight gain and reversely, children who took whole foods especially dairy products, fruits and vegetables, and grains were lower overweight or obese ,^27^.

The rate of ow/ob is greater in the first child ,^29^. In contrast, we observed a higher prevalence of ow/ob in the second child compared to the first one; this may be due to cultural and social differences across communities. Consistent with our findings, Ochiai et al. showed higher rates of obesity in younger siblings and expressed higher risks of overweight or obesity in the only child ^30^. We also found an association between lower family size, one to four members, and more prevalence of obesity; however, this relationship was not statistically significant (Table 2).

Poor cooperation of parents and the child’s caregivers and difficulties in sampling were the limitations of this study. Another limitation was missing of some factors such as parental obesity, effective determinants in childhood weight status. We suggest a surveillance study to monitor childhood obesity in order to reveal its trend and main causes.

## Conclusions

The prevalence of overweight and obesity in the study population was in a remarkable level. Therefore, national programs and strategies are required to prevent and manage obesity and its complications. If these programs start at childhood, as early as possible, considering the related risk factors, they can be more effective. In this study, the male gender, urbanization, type of father’s occupation, higher level of mother’s education, higher birth weight, second siblings, lack of using health-care services, more access to household facilities and food groups were determined as risk factors of overweight and obesity.

## Acknowledgments

The present article was financially supported by Shiraz University of Medical Sciences, Shiraz, Iran and Research Consultation Center of the University for Editorial Assistance. In addition, we are grateful to the health services’ staff in Fars Province and participating families.

## Conflict of interest statement

The authors have no conflict of interest to declare.
